# Editorial: Psychophysiology of Stress

**DOI:** 10.3389/fpsyg.2022.896773

**Published:** 2022-04-07

**Authors:** Vicente Javier Clemente-Suárez, Pantelis T. Nikolaidis, Beat Knechtle, Pablo Ruisoto

**Affiliations:** ^1^Faculty of Sports Sciences, Universidad Europea de Madrid, Madrid, Spain; ^2^Grupo de Investigación en Cultura, Educación y Sociedad, Universidad de la Costa, Barranquilla, Colombia; ^3^School of Health and Caring Sciences, University of West Attica, Athens, Greece; ^4^Department of Health Psychology, Public University of Navarre, Pamplona, Spain; ^5^Institute of Primary Care, University of Zurich, Zurich, Switzerland

**Keywords:** physiology, psychology, education, disease, sport, environmental

Stress is a multifactorial complex phenomenon where organic resources are mobilized to deal with a real or perceived threat (Cohen et al., 1983). The stress response is one of the most important phylogenetic coping mechanisms that have allowed humans to successfully adapt to highly demanding and potentially dangerous contexts (Hadany et al., 2006; Korzan and Summers, 2021).

The intrinsic neurobiological mechanisms involved in the stress response have not changed much in the last stages of the evolution of the human being, prominently including the hypothalamic-pituitary-adrenocortical axis and the autonomic nervous system (Ulrich-Lai and Herman, 2009; McEwen et al., 2015; Cohen et al., 2016). In contrast, our social context has changed dramatically recently in evolutionary terms. As result, we face a mismatch between adaptative psychophysiological stress response to acute physical stressors and the psychosocial and chronic nature of nowadays Western stressors (Cohen et al., 2016). For instance, physical activity levels continuously decreased during the last decades in the light of recent technological advances and increasingly sedentary lifestyle (Madore et al., 2020), whereas the positive role of physical activity on stress-related disorders has been widely acknowledged (Li et al., 2019; Loprinzi and Frith, 2019). Similarly, an increasing amount of studies have reported a positive correlate Western diet to acne, obesity, diabetes, heart disease and cancer, increasingly considered diseases of our civilization (Curry, 2013; Clatici et al., 2018). Moreover, exercise may play a protector role against brain aging and neurodegeneration (De Miguel et al., 2021). Indeed, socially induced chronic stress induces neuroplasticity in central stress-processing networks, causing sensitization as well as habituation of HPA axis and ANS responses with important health implications (Ulrich-Lai and Herman, 2009).

In the present special issue, it is shown how direct social evaluation of multitasking is a more potent stressor than multitasking with indirect evaluation, and the period of anticipation of stressful events may be critical to understanding the process of stress regulation. In this line and applied to the educational context, it was found how the order of presentation of different complexity/difficulty scenarios affects the autonomic stress response of undergraduate Psychology students undergoing an objective structured clinical examination. Students who underwent a high-complexity scenario first reported significantly higher autonomic stress response than students who began the objective structured clinical examination with a low-complexity scenario. Finally, in a Type 2 Diabetes older adults' population, a psychosocial stressor (Trier Social Stress Test) triggered significant increases in negative affect, cortisol and salivary alpha-amylase levels, and impaired working memory.

The present Research Topic consisted of seven research articles from different research groups and countries, covering a wide range of aspects and populations in this field. Although these articles did not cover all aspects of psychophysiology of stress, they provided important insight concerning populations that were under-represented in previous studies (e.g., students, older people with type 2 diabetes and health care professionals). Following Hariri and Holmes (2015) we hope that this collection of articles stimulates further future research in psychophysiology of stress and its implications for tackling stress-related disorders and diseases of civilization from a more comprehensive and evidence-based approach.

## Author Contributions

All authors listed have made a substantial, direct, and intellectual contribution to the work and approved it for publication.

## Conflict of Interest

The authors declare that the research was conducted in the absence of any commercial or financial relationships that could be construed as a potential conflict of interest.

## Publisher's Note

All claims expressed in this article are solely those of the authors and do not necessarily represent those of their affiliated organizations, or those of the publisher, the editors and the reviewers. Any product that may be evaluated in this article, or claim that may be made by its manufacturer, is not guaranteed or endorsed by the publisher.
